# Argonaute 2 Controls Antiviral Activity against Sweet Potato Mild Mottle Virus in *Nicotiana benthamiana*

**DOI:** 10.3390/plants10050867

**Published:** 2021-04-26

**Authors:** Erzsébet Kenesi, Juan-Jose Lopez-Moya, László Orosz, József Burgyán, Lóránt Lakatos

**Affiliations:** 1 Biological Research Center Szeged, Institute of Plant Biology, Photo- and Chronobiology Group Eötvös Loránd Research Network (ELKH), H-6726 Szeged, Hungary; kenesi.erzsebet@brc.hu; 2 Centre for Research in Agricultural Genomics (CRAG), CSIC-IRTA-UAB-UB, Campus UAB Bellaterra, 08193 Barcelona, Spain; 3 Consejo Superior de Investigaciones Científicas (CSIC), 08003 Barcelona, Spain; 4 Department of Medical Microbiology and Immunobiology, University of Szeged, H-6720 Szeged, Hungary; orosz.laszlo@med.u-szeged.hu; 5 Agricultural Biotechnology Institute, National Agricultural Research and Innovation, H-2100 Gödöllő, Hungary; burgyan.jozsef@mbk.naik.hu

**Keywords:** RNA silencing, AGO1, AGO2, sweet potato mild mottle virus

## Abstract

RNA silencing is a sequence specific post-transcriptional mechanism regulating important biological processes including antiviral defense in plants. Argonaute (AGO) proteins, the catalytic subunits of the silencing complexes, are loaded with small RNAs to execute the sequence specific RNA cleavage or translational inhibition. Plants encode several AGO proteins and a few of them, especially AGO1 and AGO2, have been shown to be required for antiviral silencing. Previously, we have shown that the P1 protein of the sweet potato mild mottle virus (SPMMV) suppresses the primary RNA silencing response by inhibiting AGO1. To analyze the role of AGO2 in antiviral defense against the SPMMV, we performed a comparative study using a wild type and ago2^−/−^ mutant *Nicotiana benthamiana*. Here we show that the AGO2 of *N. benthamiana* attenuates the symptoms of SPMMV infection. Upon SPMMV infection the levels of AGO2 mRNA and protein are greatly increased. Moreover, we found that AGO2 proteins are loaded with SPMMV derived viral small RNAs as well as with miRNAs. Our results indicate that AGO2 protein takes over the place of AGO1 to confer antiviral silencing. Finally, we provide a plausible explanation for the AGO2 mediated recovery of an SPMMV-infected sweet potato.

## 1. Introduction

RNA silencing refers to one of the cellular pathways that regulates gene expression through specific mechanisms acting on RNA and is based on the sequence specificity of nucleic acids. RNA silencing is considered to have evolved to regulate gene expression both in the nucleus and the cytoplasm of almost all eukaryotes. Among many functions, RNA silencing serves as an antiviral mechanism in plants. The replicative forms of ssRNA viruses or other sources of dsRNA such as regions with strong secondary structures result in double-stranded RNA that triggers RNA silencing. This viral dsRNA is recognized and processed into viral siRNAs (vsRNAs) by the Dicer-like (DCL) enzymes. vsRNAs are then associated with argonaute (AGO) proteins, which are the central molecule of the RNA induced silencing complex (RISC) and guide the RISC to the viral nucleic acids for target cleavage or translational inhibition [1].

Plant viruses evolved viral suppressors of RNA silencing (VSR) proteins to counteract RNA silencing. VSRs are very diverse proteins and inhibit different steps of RNA silencing such as the generation of vsRNAs, the sequestering of vsRNAs, the formation of RISC complexes or the inhibition of a pre-assembled RISC [1,2].

The genome of *Arabidopsis thaliana* encodes 10 AGO proteins that are shown to be involved in different RNA silencing pathways and AGO1 was first shown to be required for antiviral defense [3]. Hypomorphic AGO1 mutants are more vulnerable to brome mosaic virus (BMV, genus *Bromovirus*, family *Bromoviridae*) and the cucumber mosaic virus (CMV, genus *Cucumovirus*, family *Bromoviridae*) relative to the wt *A. thaliana* [4,5,6]. However, the AGO1 mutant is less susceptible to the tobacco rattle virus (TRV, genus *Tobravirus*, family *Virgaviridae*) [7]. 

AGO2 is required for antiviral defenses against a broad range of viruses including the turnip crinkle virus (TCV, genus *Betacarmovirus*, family *Tombusviridae*), the TRV, potato virus X (PVX, genus *Potexvirus*, family *Alphaflexiviridae*), the turnip mosaic virus (TuMV, genus *Potyvirus*, family *Potyviridae*) and the tomato bushy stunt virus (TBSV, genus *Tombusvirus*, family *Tombusviridae*) [8,9,10,11,12]. However, the lack of AGO2 had a subtle effect against the cymbidium ringspot virus (CyMRSV, genus *Tombusvirus*, family *Tombusviridae*) and the CMV [13]. Therefore, it is widely accepted that AGO1 and AGO2 proteins act as the first line of antiviral RNA silencing in the case of certain viruses. 

The sweet potato mild mottle virus (SPMMV; *genus Ipomovirus, family Potyviridae*) is a positive single stranded RNA virus with a genome of about 10,000 nucleotides (nt) in length encoding a polyprotein of about 3450 amino acids and an embedded out-of-frame shorter additional gene product. The genome organization and the length of the mature proteins are very similar to that of potyviruses [14]; however, the P1 protein of the SPMMV differs most from the corresponding proteins of other *Potyviridae* [15,16].

Previously we found that the P1 VSR protein of the SPMMV physically interacts with AGO1 containing pre-assembled RISC complexes [17]. Using an agrobacterium-based infiltration system to transiently express in vivo different gene products we could measure specific AGO activity and we revealed that P1 inhibits the target cleavage of AGO1. Whilst P1 interacts with AGO2, the target cleavage activity was not affected by P1 [18]. Therefore, it is an interesting question to answer whether beyond AGO1, AGO2 takes part in the antiviral defense against the SPMMV in vivo.

## 2. Results

### 2.1. The N. benthamiana AGO2^−/−^ Mutant Is More Susceptible to the SPMMV Compared with Wild Type Plants

**Figure 1:**
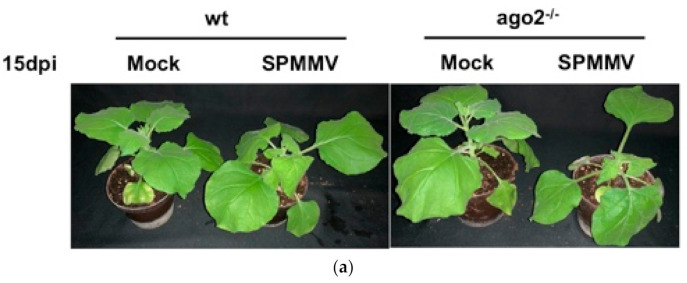

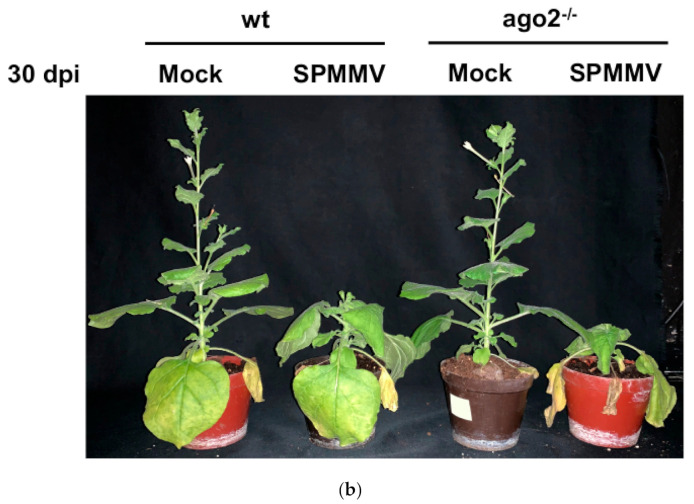
(**a**) AGO2 deficiency exacerbates symptoms of SPMMV infection in *N. benthamiana*. Wt and AGO2^−/−^ *N. benthamiana* plants were infected with the sap of SPMMV-infected *N. benthamiana* leaves. As controls, mock infections were also performed. Photographs of the SPMMV and mock-infected wild type and AGO2^−/−^ plants were taken at (**a**) 15 and (**b**) 30 dpi.

To test whether AGO2 plays a role in antiviral defense against the SPMMV, wild type (wt) and AGO2^−/−^ mutant *N. benthamiana* plants were infected with the SPMMV. The predominant symptoms of the SPMMV-infected plants were leaf curling and mottling developed in systemic leaves. SPMMV infection also caused stunting in plants (Figure 1a,b). Moreover, the SPMMV-infected AGO2^−/−^ mutant *N. benthamiana* plants had more distorted leaves, more pronounced mottling and more severe stunting than the infected wt plants at both 15 and 30 dpi.

**Figure 2:**
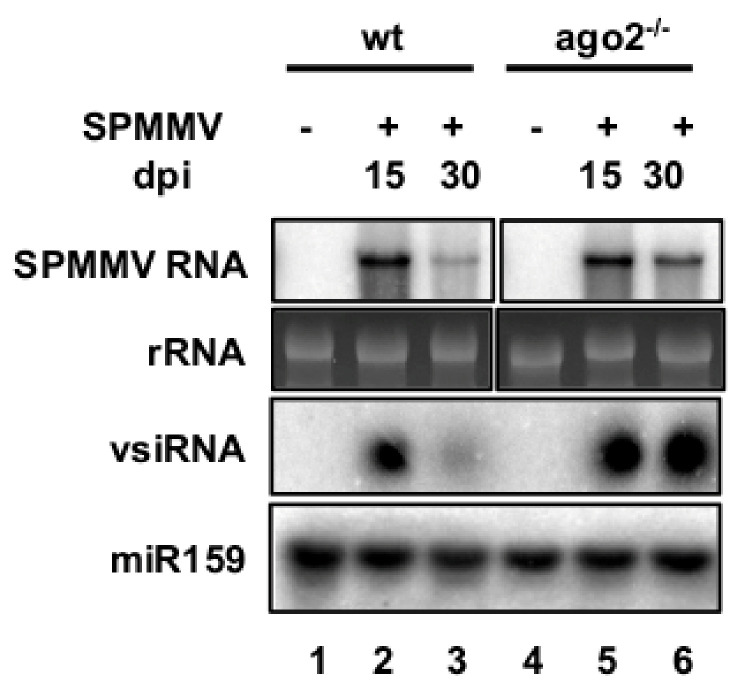
Northern analysis of SPMMV-infected plants. RNA extracts of SPMMV-infected wt and ago2^−/−^ at 15 and 30 dpi and mock-infected plants were subjected to a Northern analysis using denaturing agarose gels. rRNA was used for the loading control. The same RNA extracts were separated on a 10% acrylamide and 8 M urea containing denaturing gel to detect small RNAs. A negative strand [α-32P]-UTP-labeled RNA probe was used to detect the viral genomic RNA and vsRNAs. A [γ-32P]-ATP-labeled LNA oligo to detect miR159 was used to show equal loading.

To find out whether the severity of symptoms was correlated with the SPMMV level we used Northern blot hybridization. Our analysis revealed a higher amount of SPMMV genomic RNA at 15 dpi than at 30 dpi in the wt plants. In *N. benthamiana* we detected a slight difference in the abundance of genomic RNA. However, the RNA extracts from the AGO2^−/−^ mutant plant at 15 dpi contained more genomic RNA than that of the extracts from the SPMMV-infected mutant at 30 dpi. Finally, the abundance of SPMMV derived small RNAs was consilient with the genomic RNA level in all samples (Figure 2).

### 2.2. SPMMV Infection Induces AGO2

**Figure 3:**
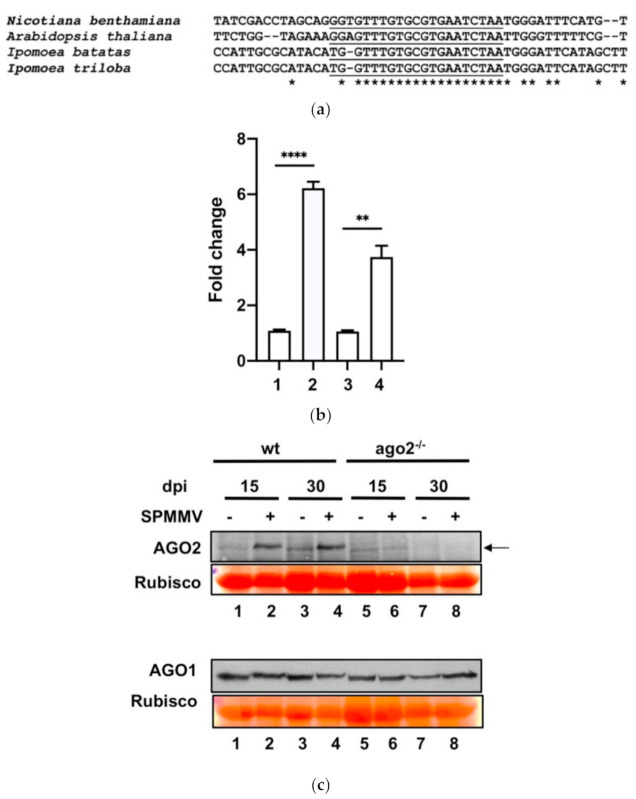
AGO2 mRNA and protein expression is induced by SPMMV infection. (**a**) Clustal sequence alignment of the 3′ end UTR containing the miR430 target site of *N. benthamiana* (read identifier Niben101Scf05245g01007.1), *A. thaliana* (NM_102866.3), *Ipomoea batatas* (read identifier gnl|SRA|SRR7866901.255798333) and *Ipomoea triloba* (XM_031274565.1). The * indicates nucleotide identity. (**b**) *N. benthamiana* plants were infected with the SPMMV then RNA samples were taken at 15 and 30 dpi (1-mock treated at 15 dpi; 2-SPMMV-infected at 15 dpi; 3-mock treated at 30 dpi; 4-SPMMV-infected at 30 dpi). AGO2 mRNA expression was normalized to that of the endogenous elongation factor 1 (EF1). A mock-infected control sample was used as the calibrator and three independent biological replicates of each treatment were carried out. For each biological replicate, two parallel samples were analyzed. The * indicates statistically significant differences between the mock and infected plants at 15 and 30 dpi according to a two-sample T-test Bonferroni post-hoc test (** *p* < 0.001; **** *p* < 0.0001). (**c**) Leaf samples of the SPMMV and mock-infected wt and AGO2^−/−^ *N. benthamiana* plants were taken at 15 and 30 dpi and protein extracts were analyzed by SDS-PAGE followed by incubation with the AGO2 antibody (19). As a control, the same protein extracts were analyzed on a separate gel to detect AGO1 (Agrisera). Ponceau staining was used as a loading control. The arrow indicates a non-specific band in the mock-infected wt and AGO2^−/−^ mutant *N. benthamiana* plants.

Using the well-established system based on agroinfiltration to measure specific AGO activity [11], we recently showed that mock infiltrated *N. benthamiana* leaves did not possess AGO2 activity. However, the sole administration of the SPMMV P1 protein by agroinfiltration strongly induced AGO2 target cleavage activity and mRNA levels [18]. As SPMMV P1 VSR inhibited AGO1 miRNA and vsRNA driven target RNA cleavage [17] and because the *N. benthamiana* (as well as *A. thaliana*, *Ipomoea batatas* and *Ipomoea triloba*) AGO2 mRNA contains one miR403 binding site in the 3′ untranslated region (UTR) (Figure 3a), we speculated that the global inhibition of AGO1 mediated miRNA silencing by P1 would liberate AGO2 mRNA from silencing [18].

To see if AGO2 induction occurred in vivo, we assessed AGO2 mRNA and the protein expression in SPMMV-infected wt *N. benthamiana* plants. RNA was isolated from the systemic leaves of the SPMMV and mock inoculated *N. benthamiana* plants at 15 and 30 dpi and subjected to qRT-PCR to measure the AGO2 mRNA level. Our analysis revealed a significant increase of AGO2 mRNA upon SPMMV infection at both 15 and 30 dpi (Figure 3b); however, the fold change was more pronounced at 15 dpi than at 30 dpi (6.22 vs. 3.74). A Western blot analysis with an AGO2 specific antibody of mock and SPMMV-infected wt *N. benthamiana* plants detected a ~110 kDa protein similar to the estimated size of the *A. thaliana* AGO2 protein [8] in the protein extracts of the virus-infected plants at both 15 and 30 dpi (Figure 3c; lanes 1 and 2; 3 and 4). As a control, we used homozygous AGO2^−/−^ mutant *N. benthamiana* plants that do not express AGO2. As expected, we could not detect this 110 kDa protein in the extracts of the mock and SPMMV-infected AGO2^−/−^ mutant *N. benthamiana* plants (Figure 3c; lanes 1, 2 or 3, 4 to 5–8)

Therefore, we concluded that SPMMV infection strongly elevated endogenous AGO2 expression at both the mRNA and protein level in wt *N. benthamiana* leaves.

### 2.3. AGO2 Associates with Viral siRNAs and miRNAs in SPMMV-Infected Plants

**Figure 4:**
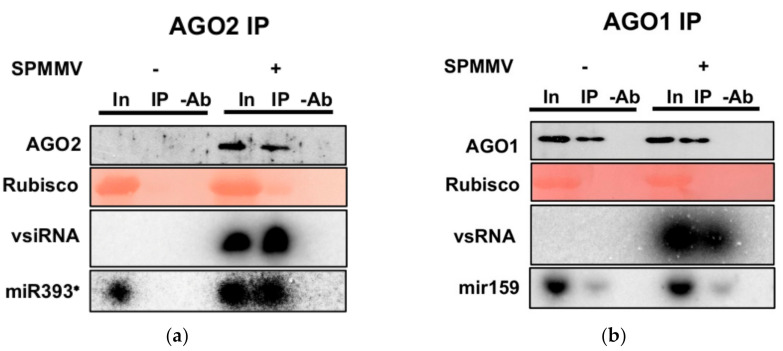
AGO2 associates with vsRNAs and miRNAs. (**a**) SPMMV and mock-infected plants at 15 dpi were used to prepare native protein extracts. Immunoprecipitations were carried out with the AGO2 antibody. One half of the IP fractions was used to isolate proteins while the other half was used for RNA preparation. Input, IP and control IP with no antibody (-Ab) samples were used to detect AGO2 proteins by Western blotting; vsRNAs and miRNAs were detected by Northern blotting using a [α-32P]-UTP-labeled single stranded in vitro transcribed RNA probe and a DNA oligo to detect miR393*. (**b**) This panel was carried out as in (**a**), but the AGO1 antibody was used to immunoprecipitate and detect the AGO1 protein. For Northern blotting an [γ-32P]-ATP-labeled miR159 LNA oligo was used.

The more severe symptoms upon SPMMV infection on homozygous AGO2^−/−^ mutant compared with the wt *N. benthamiana* plants and the inducible nature of AGO2 highly suggested the involvement of AGO2 in the antiviral defense against the SPMMV. The nature of small RNAs associated with a few certain AGO proteins is a good indicator of their function [1,4,8,19,20]. Thus, the association of viral siRNA with AGO2 of *N. benthamiana* would provide further evidence of an antiviral function. To test if *N. benthamiana* AGO2 protein binds SPMMV derived siRNAs, we performed immunoprecipitation followed by Western and Northern blotting from mock and SPMMV-infected plants. As we previously showed, AGO2 was induced by SPMMV infection (Figure 3b,c) and the input and eluate fractions of SPMMV-infected wt plants contained vsRNAs providing strong evidence for a physical interaction with the AGO2 protein (Figure 4a; lanes 4 and 5). As expected, no vsRNA was detected in the mock-infected plants (Figure 4a; lanes 1–3).

The pivotal role of AGO1 in antiviral defense has been long known [1]. In our host-pathogen system we found similar AGO1 expression in *N. benthamiana* irrespective of SPMMV infection and genetic background (wt vs. AGO2^−/−^ mutant) (Figure 4b). Therefore, AGO1 was also immunoprecipitated from the mock and SPMMV-infected leaves of *N. benthamiana*. As expected, vsRNAs could be pulled down with AGO1 only from native extracts of SPMMV-infected plants (Figure 4b; lanes 4 and 5) and we did not detect AGO1 protein co-immunoprecipitated with vsRNAs from the uninfected leaves of *N. benthamiana* (Figure 4b; lanes 1 and 2). To further validate our results, we took advantage of the differential sorting of plant miRNAs to AGO1 and AGO2 proteins [21]. For example, miR393* (the star strand of the miR393 duplex) was shown to be loaded to AGO2 while miR159 was almost exclusively found in association with the AGO1 protein [20]. In agreement with previous results, we found miR393* expression in the inputs of both the mock and SPMMV-infected *N. benthamiana* leaves but miR393* could be only pulled down from SPMMV-infected plants in which AGO2 protein was induced (Figure 4a; lanes 4 and 5), further proving that AGO2 was induced and specifically associated with miRNAs as well. Finally, in the eluates of AGO1 immunoprecipitations miR159 was detected in both the mock and SPMMV-infected *N. benthamiana* plants, indicating that miRNAs were associated with AGO1 regardless of virus infection (Figure 4b; lanes 1, 2 and 4, 5).

## 3. Discussion

The genome of higher plants contains several AGO genes. For example, the genome of the model plant *Arabidopsis thaliana* encodes 10 AGO proteins that are specialized to several RNA silencing pathways [3,22]. A genetic analysis revealed a possible involvement of AGO1, AGO2, AGO4, AGO5, AGO7 and AGO10 in antiviral defense [11,23,24]. However, AGO1 and AGO2 are thought to be the main AGO proteins involved in antiviral defense in *A. thaliana* and other plant species [23].

Our goal was to elucidate the role of AGO2 in the widely used test plant *N. benthamiana* upon SPMMV infection. We observed that the homozygous AGO2^−/−^ null mutant *N. benthamiana* plants were more susceptible to SPMMV infection than that of the wild plants implicating the role of AGO2 in the antiviral defense. In contrast SPMMV infection of the sweet potato plants, the natural host of the SPMMV, exhibited mild vein chlorosis and mottling for 2–4 weeks then plants recovered from the infection [25]. The difference in symptomatology between the two species might be explained by the fact that wt *N. benthamiana* is more prone to RNA viruses than other species [26].

In the model plant *A. thaliana*, AGO1, AGO2 and AGO5 have been shown to bind virus derived vsRNAs in an immunoprecipitation analysis [24]. Recently, it was found that AGO2 mutant *A. thaliana* plants were hypersusceptible to the TCV and the CMV [8]. Moreover, AGO2 was also absolutely required to control PVX infection in Arabidopsis [9].

In agreement, the AGO2 null mutant *N. benthamiana* was hypersusceptible to the TCV, PVX and the TuMV [13]. However, the same study revealed that AGO2 had little effect to control CMV and CymRSV infection in *N. benthamiana* suggesting that AGO1 but not AGO2 might have a critical role in coping with CymRSV and CMV infection. In contrast, AGO2 silenced *N. benthamiana* plants with TBSV (a close relative of the CymRSV) infection caused severe symptoms [10]. 

In conclusion, plant RNA viruses show different sensitivity to AGO2 mediated RNA silencing yet the AGO2 protein is required to restrict a broad range of viruses including the SPMMV.

Using a transient expression method based on infiltration, specific AGO1 and AGO2 target cleavage activity could be measured. With this technique, we recently revealed that normal *N. benthamiana* leaves do not possess AGO2 activity. However, a transient expression of the P1 silencing suppressor of the SPMMV by itself increased the mRNA and target cleavage activity of AGO2 [18]. In agreement, upon SPMMV infection (this study) both AGO2 mRNA and the protein expression were strongly upregulated in *N. benthamiana* leaves. As we showed earlier, the P1 silencing suppressor of the SPMMV efficiently inhibited (viral siRNA) vsRNA and miRNA induced RNA silencing by binding the AGO1 protein of RISC complexes. Therefore, the liberation of AGO2 mRNA from miR403-AGO1 mediated repression provided a plausible explanation for the strong induction of AGO2 mRNA and protein expression upon P1 infiltration and SPMMV infection [17,18] (this study). Our results were further supported by the fact that the AGO1–25 *A. thaliana* mutant, in which the miR403-AGO1 mediated repression of AGO2 mRNA does not occur, was constitutively expressing AGO2 [8].

The target cleavage and translational repression by RNA silencing requires the formation of AGO-small RNA complexes. In SPMMV-infected plants we detected SPMMV derived vsRNAs co-immunoprecipitated with AGO2 suggesting that these ribonucleoprotein complexes could serve as functional RISC complexes. Thus, the more severe symptoms of the SPMMV-infected AGO2^−/−^ mutant *N. benthamiana* compared with that of the wt suggested that the AGO2-vsRNA complex was a critical component of the antiviral defense against the SPMMV. Plant AGO2 was also found to take part in miRNA driven RNA silencing. In agreement, we detected miR393*, which inhibits retrograde transport in *Pseudomonas*-infected *A. thaliana*, in our immunoprecipitation from SPMMV-infected leaf extracts indicating that functional miR393*-AGO2 complexes were formed upon viral infection [20] (this work). 

Besides the miR403-AGO1 driven RNA silencing, other ways of regulating AGO2 expression have been found in virus-infected plants. For example, upon tomato ringspot virus-Rasp1 (ToRSV-Rasp1, genus *Nepovirus*, family *Secoviridae*) and ToRSV-GYV infection, a strong and transient induction of AGO2 mRNA was detected leading to AGO2 protein production [27]. The authors speculated that the transient AGO2 induction occurred at the level of transcription. However, in this host-pathogen system, the de novo translated AGO2 protein was then downregulated by cellular protein degradation mechanisms in systemic leaves in infected plants indicating the possible dual regulation of AGO2 [27]. Another study revealed that upon TuMV infection, the mature form of miR403 was induced (and consistently the pri-miR403 level was reduced). Moreover, AGO2 mRNA was strongly induced suggesting that upregulation could escape AGO2 mRNA from AGO1-miR403 repression [28]. Indeed, gibberellic acid, UV irradiation and bacterial infection upregulated AGO2 mRNA revealing that AGO2 was regulated by a variety of biotic and abiotic stresses [20,29,30].

Our results on *N. benthamiana* point out the critical role of AGO2 in the antiviral defense against the SPMMV. The results presented here allow us to postulate a model for infection of *N. benthamiana* by the SPMMV. SPMMV infection results in viral P1 protein and vsRNA production. Activity of the de novo AGO1-vsRNA complexes might be neutralized by the P1 protein. As the association of P1 with AGO1-miRNA pre-assembled RISC complexes inhibits miRNA (including miR403) driven RNA silencing, AGO2 mRNA could escape repression by AGO1-miR403 leading to AGO2 protein production. Thus, AGO2 might take over the place of AGO1 and being expressed in infected cells could provide the necessary and sufficient RNA silencing activity that leads to attenuate symptoms in *N. benthamiana*. This model is supported by our recent results that the physical interaction between AGO2 and P1 proteins did not lead to the inhibition of the target cleavage activity of AGO2 [18]. Of note was that AGO1-miR403 regulation might occur in a sweet potato as well because the 3′ UTR of the AGO2 mRNA also contains an miR403 target site therefore our model on *N. benthamiana* could be extended to a sweet potato. As symptoms upon SPMMV infection on a sweet potato are milder than that of *N. benthamiana*, it could not be excluded that a defense mechanism other than miR403-AGO1 driven RNA silencing might be involved in antiviral defense against the SPMMV in a sweet potato.

## 4. Materials and Methods

### 4.1. Plant Materials

*Nicotiana benthamiana* wt and AGO2^−/−^ mutant plants were grown at 23 °C in a plant growth chamber under a photoperiod of 16 h light/8 h dark.

### 4.2. Plant Inoculation

Sap from the SPMMV 130 isolate (originally from Tanzania, partial sequence available at GenBank: GQ353374.1) was obtained. The infected leaves were freshly extracted in a phosphate buffer of pH9.0 and supplemented with carborundum powder and were used to inoculate 18 day old *N. benthamiana* plants. Samples were collected at 15 and 30 dpi.

### 4.3. RNA Analysis

RNA was isolated using the 2 × PK buffer (200 mM Tris-HCl pH8.0, 300 mM NaCl, 20 mM EDTA pH8.0) supplemented with 10 μg/mL proteinase K. The reactions were incubated at 55 °C for 10 min then extracted with phenol-chloroform and nucleic acids were precipitated with 2 volumes of EtOH. The RNA was separated by 2.2 M formaldehyde and 1.2% *w/v* agarose gels and blotted to an Amersham Hybond-N membrane. The membranes were hybridized with 500 base [α-32P]-UTP-labeled single stranded in vitro transcribed RNA probes corresponding with the negative strand of the P1 coding region of the SPMMV genome.

### 4.4. Small RNA Analysis

RNA was isolated with a 2 × PK buffer (200 mM Tris-HCl pH8.0, 300 mM NaCl, 20 mM EDTA pH8.0) supplemented with 10 μg/mL proteinase K from leaves or the eluates of immunoprecipitations and then separated on a 10% acrylamide and 8 M urea containing denaturing gel and blotted to an Amersham Hybond-N membrane. The membranes were hybridized with [γ-32P]-ATP-labeled LNA or DNA oligonucleotides. 

### 4.5. Protein Analysis

Proteins were isolated using the 4 M urea, 50 mM Tris-HCl, 100 mM NaCl and 20 mM EDTA pH8.0 containing buffer. A total of 20 μg protein was loaded to a 6% or 10% acrylamide SDS-PAGE gel then transferred to an Immobilon-P membrane (Millipore). The membranes were incubated with AGO1 (Agrisera) or AGO2 antibodies (19) in a TBS-TT buffer (50 mM Tris-HCl, pH7.6, 150 mM NaCl, 0.25% Tween 20 and 0.1% Triton X-100).

### 4.6. Immunoprecipitation

Extracts were prepared in an IP buffer (50 mM Tris-HCl pH7.5, 100 mM NaCl, 5 mM MgCl_2_, 5 mM DDT and 0.5% Tween 20) and were incubated with AGO1 (Agrisera) or AGO2 antibodies [19]. Eluates were used to isolate AGO-small RNA complexes. For inputs, 5% of the native extracts and 50% of the eluates were used to isolate proteins or small RNAs. 

### 4.7. Quantitative Reverse Transcriptase PCR (qRT-PCR)

RNA was isolated with a Trizol reagent (Sigma) from mock and infected systemic leaves at 10 dpi. qRT-PCR experiments were performed as described by [18].

## Data Availability

Not applicable.
